# Development of competency model for family physicians against the background of ‘internet plus healthcare’ in China: a mixed methods study

**DOI:** 10.1186/s12960-020-00507-6

**Published:** 2020-09-11

**Authors:** Ziling Ni, Xiaohe Wang, Siyu Zhou, Tao Zhang

**Affiliations:** 1 Department of Social Medicine and Health Service Management, School of Medicine and Health Management, Hangzhou Normal University, NO. 2318, Yuhangtang Rd, Yuhang District, Hangzhou, Zhejiang People’s Republic of China; 2 Department of Health Management, School of Medicine and Health Management, Tongji Medical College, Huazhong University of Science and Technology, No. 13 Hangkong Road, Wuhan, 430030 Hubei People’s Republic of China

**Keywords:** Family physician, Service competences, Assessment tool, Internet plus healthcare

## Abstract

**Background:**

Identification of the service competences of family physicians is central to ensuring high-quality primary care and improving patient outcomes. However, little is known about how to assess the family physicians’ service competences in primary care settings. It is necessary to develop and validate a general model of core competences of the family physician under the stage of construction of family doctor system and implementation of ‘Internet Plus Healthcare’ service model in China.

**Methods:**

The literature review, behavioural event interviews, expert consultation and questionnaire survey were performed. The scale’s 35 questions were measured by response rate, highest score, lowest score, and average score for each. Delphi method was used to assess content validity, Cronbach’s *α* to estimate reliability, and factor analysis to test structural validity. Respondents were randomly divided into two groups; data for one group were used for exploratory factor analysis (EFA) to explore possible model structure. Confirmatory factor analysis (CFA) was then performed.

**Results:**

Effective response rate was 93.56%. Cronbach’s *α* coefficient of the scale was 0.977. Factor analysis showed KMO of 0.988. Bartlett’s test showed *χ*^2^ of 22 917.515 (df = 630), *p* < .001. Overall authority grade of expert consultation was 0.80, and Kendall’s coefficient of concordance *W* was 0.194. By EFA, the five-factor model was retained after thorough consideration, and four items with factor loading less than 0.4 were proposed to obtain a five-dimension, 32-item scale. CFA was performed on the new structure, showing high goodness-of-fit test (NFI = 0.98, TLI = 0.91, SRMSR = 0.05, RMSEA = 0.04). Overall Cronbach’s *α* coefficients of the scale and each sub-item were greater than 0.9.

**Conclusions:**

The scale has good reliability, validity, and credibility and can therefore serve as an effective tool for assessment of Chinese family physicians’ service competences.

## Introduction

Facing with the challenges posed by ageing and increasing global healthcare demand, there has been an increase in the emphasis placed on the importance of primary care models that can provide continuous, coordinated, and comprehensive care services for individuals and families [1]. As the comprehensive health service provider in communities, family physicians have played a critical role in public health worldwide [2].The family doctor system is also the most effective approach to achieving universal health coverage and the foundation of health system globally [3, 4]. Being different with specialists who practice in hospitals, family physicians often manage patients’ health in teams, providing a full-life primary healthcare service including basic medical care, establishment and management of health records, health management of patients with chronic diseases, children’s vaccination, maternal healthcare, and prevention and control of infectious diseases (e.g. tuberculosis, AIDS) [5, 6]. Therefore, family physicians have to have comprehensive medical knowledge and can provide comprehensive, continuous, timely, personalized medical services [7, 8].

The family doctor system in developed countries such as the United Kingdom, the United States of America, Canada, Australia, and Japan has well developed [2, 9]. The system of ‘health service gatekeeper’ have provided useful experience for the Chinese medical reform. In response to the challenges of an ageing population, high incidence of chronic diseases, and rising medical costs, the Chinese government has been implementing strategies to strengthen primary health care: establishing a primary medical care service infrastructure and driving the development of a family doctor system. In 2011, the State Council (the main national administrative government body) proposed *Guiding Opinions on Establishing a General Practitioner System*, following the *Notice on Issuing and Pushing Forward the Guiding Opinions on Promoting Family Physicians’ Contract Services* in 2016. These policies put forward new requirements for primary health care systems, especially family doctors. It is expected that family physicians who serve as health gatekeepers for residents are mainly working at grassroots medical institutions, such as community health centres and village health facilities. Alongside the development of family physician system, the government is also promoting application of health information technology (HIT) in family doctor services (electronic health records, online medical consultation, remote consultation, and medical information inquiry) as an important part of ‘Internet Plus Healthcare’ plan in China. According to the Healthy China 2030 Blueprint released by the Party Central Committee and the State Council in October 2016, efforts will be made to foster new industries, new forms and models of business in the health sector and to develop Internet-based health services to promote ‘Internet Plus Healthcare’ [10]. Implementation and application of electronic health records (EHRs) and other HIT has been shown to facilitate primary care practice transformation, which has changed service delivery and led to higher requirements for service competences of family physicians [5, 6]. Therefore, family physicians face the dual challenge of increasing service volume and changing the service model. In China, however, due to the short history of primary care doctors, primary care is a relatively new discipline in medical schools in China. Many family doctors have been transferred form hospital specialists and need appropriate training in family medicine or general practice. In addition, identification of the service competences of family physicians are central to ensuring high-quality primary care and improving patient outcomes [11, 12]. Scientific evaluation of family physicians’ service competences is a necessary preliminary to improving them, and a reliable, credible, valid evaluation scale is therefore essential [13]. It can help primary healthcare managers to develop targeted training and improve incentive mechanism for family physicians. Some studies noted that tools to identify the competencies for family physicians required to address the local community’s health needs should be developed [3, 14].

Although several survey tools exist to measure a particular aspect of a family doctor’s ability based on replicating ideas from high-resource settings (e.g. communication skills, diagnostic ability) [15, 16], rather than comprehensive ability that meet the community’s needs. Existing methods of assessing family doctors’ ability are also not targeted toward family doctors in particular [17–19]. Therefore, this study proposes a conceptual framework to evaluate service competences of family physicians in the context of developing ‘Internet Plus’ family doctor service in China. Based on a literature review, assessment scales applicable to Chinese family physicians’ service competences are developed and empirically validated.

## Methods

### Scale development

Initial competence assessment items in four-dimensional framework were developed in three phases. First, based on Competency Onion Model [20], a comprehensive literature review was performed. Web of Science Core Collection, MEDLINE, and PubMed databases and China National Knowledge Infrastructure (CNKI) were searched, and Google search engine was further employed using key words of family medicine, family physician, family doctor, competence, competency, and competency model for the period of January 2000 to December 2017, both in English and Chinese. The searching strategy also included manual search of journals, grey literature, and references of included articles. Inclusion criteria were as follows: the content of the article is related to the competence/ability/performance/skills of the family physician. Exclusion criteria were as follows: being presented in congress, letter to editor, and case report, and not mentioning any contents about competence/ability/performance/skill. Initial searches identified 228 records, of which 207 were excluded after excluding inconsistent and duplicated cases. After excluding inconsistent and duplicated cases, 21 articles were finally included. Manual search revealed two thesis and two industry reports. The final number of assessed resources was 25. In this phase, with considering the context and content of current services provided by Chinese family physicians, the instruments used to assess family physicians’ service competency models in different countries and regions (such as Canada [21], South Africa [22], the United States of America [23], and European [24, 25]) were combed thoroughly to develop a list of four-dimension (46 items) initial competency elements of family physicians: service skills, professionalism, interpersonal communication and teamwork skills, and personal traits. Second, the achievement orientation of family physicians at different levels of performance was studied by structural interviews and behavioural event interviews (BEIs). The BEIs is a technique to identify the competencies needed to perform a job in a proper manner. The BEIs is focused on the events of interviewees as well as their opinions and concerned with the interviewees’ own insights into certain occurrences [26, 27]. Under the STAR (Situation, Task, Action, Result) principle of the BEIs [28], interviewees were asked to recall and share key examples and information of fulfilling and frustrating moments (as positive and negative outcomes, respectively) at work. Based on the principle of data saturation [29], 32 general practitioners/family physicians from Zhejiang province and Guangdong province were interviewed in depth. The positive and negative outcomes were compared separately, and then, the competency elements were extracted based on separate recordings from interviewees in excellent performance group and a general performance group. Then, the potential competency items were then modified on this basis of identifying unique traits of outstanding performance. Third, based on the literature review and interviews, two rounds of expert consultation were conducted to test the content validity (dimensions, items, language) of the scale, with 20 experts from different fields. The specialties of the experts included management, education, and scientific researchers on primary care. According to these consultations, the items were modified mostly in minor ways. All the interviews were conducted in Chinese. Both of the BEIs and expert interviews were digitally recorded and transcribed and analysed in Nvivo11.0. The items that interviewees suggested which were not applicable in the context of primary care were dropped. According to the results of interviews, we modified some items, dropped items that were not relevant, and added items that were considered important. Ultimately, a 35-item from four-factor questionnaire (Is the item important for family physician competency model?) was developed for inclusion and testing in a survey of family physicians. Each question was scored using the 5-point Likert scale (1 = strongly disagree, 5 = strongly agree).

### Sampling and data collection

A questionnaire survey was conducted with family physicians who gave their informed consent to participate. The research object was family physicians in Hangzhou and Shenzhen, where the family doctor system is well developed. Hangzhou, in eastern China, has nine administrative districts, 197 community health service centres, and more than 1180 general practitioners. The total number of residents who are signing a contact with a family physician for health services is 1.5 million. Shenzhen, in southern China, has 10 administrative districts, more than 600 community health service centres, and 3843 family doctors for a total of 3.8 million residents who are signing with a family physician. First, two districts from nine districts in Shenzhen and 10 districts in Hangzhou were selected. Second, all the 30 community health service centres in the four districts were included. Systematically, from June to December 2018, the face-to-face interviews of all family who met the inclusion criteria of the survey were conducted by four trained investigators in every community health service centre included in this study. The inclusion criteria of participants were as follows: informed consent and 1 year of work experience as family physician in community health service centre.

### Data analysis

Item analysis was used to verify and analyse responses to each question (including ceiling/floor effects and consistency/difference). Response rate, highest score, lowest score, and average score were measured for each question. Reliability, or credibility, refers to the degree of consistency or stability of measurement; in this paper, homogeneity reliability, that is, internal consistency between scale items, was used, with Cronbach’s *α*, a common reliability index for Likert results [30]. Validity refers to effectiveness: the degree to which a measuring tool or method accurately measures what is required [31]. Factor analysis (Kaiser–Meyer–Olkin (KMO) value analysis and Bartlett’s spherical test) was used to test the scale’s structural validity. In general, the closer KMO is to 1, the more the common factors among the variables, making them more suitable for factor analysis. As a supplement, Bartlett’s spherical test verified the correlation matrix of each item: a significance level of 0.05 indicates common factors among the correlation matrices of each item in the scale [32].

Respondents were randomly divided into two groups. The first group’s data were used for the EFA to explore what possible scale structure would be appropriate. Given the possibility of high correlations between various factors, the rotation method was adopted to perform orthogonal rotation processing, so that each item had a large different factor loading in each common factor, which was beneficial to identify the common factors. To ensure item differentiation, questions were selected based on the factor loading of each item in each common factor: items with factor loading of 0.4 or above were retained to ensure identification of the item, which helped distinguish scale structure more clearly. Items with factor loading less than 0.4 were excluded. Three criteria were used to determine possible dimensions: (1) eigenvalue of the factor was higher than the average eigenvalue, (2) the apparent turning point occurred in the gravel map, and (3) there were more than two items, and factor fit was above 0.4 [30].

EFA was followed by CFA, using the remaining set of samples that had been randomly selected for EFA. Chi-squared test of goodness-of-fit (*χ*^2^/df), non-normed fit index (NFI), standardized root–mean–square residual (SRMSR), and root–mean–square error of approximation (RMSEA) were selected as indexes of goodness-of-fit; NFI > 0.90, TLI > 0.90, and RMSEA < 0.08 indicate acceptable fit. Cronbach’s *α* was utilized to test internal consistency of potential factors; reliability is considered acceptable if *α* ≥ 0.70. A *p* value of < 0.05 was considered statistically significant. We performed EFA and CFA using Mplus version 8.0.01. All other analyses were performed using R 4.0.0.

## Results

### BEIs

The 20 BEIs generated 40 outcomes respectively: 20 positive results (successful events) and 20 negative results (failure events). The transcriptions in Chinese totalized 9452 words. The data were coded through highlighting passages of text according to the research questions posed. There were 125 open codes that 71 codes were extracted from positive outcomes, and 54 codes from negative outcomes. The coding results suggested that most of the elements of competence mentioned in the coding data were consistent with the results of literature review. In addition, we found that the family physicians with outstanding performance were pronounced to mention some keywords about ‘online-healthcare service’. Therefore, we added four items: ‘Online health education’, ‘Online health promotion’, ‘Online health guide’, and ‘Online information exchange’.

### Expert consultation

The experts mentioned that ‘Preventive medical service’ is a broad concept as a part of public health sciences including some items in the same dimension, such as ‘Health education’ and ‘Health management’. Therefore, experts suggested that the item ‘Preventive medical service’ should be modified to ‘Disease surveillance’.

In addition, the experts advised that item of ‘understanding and implementation of primary healthcare regulations’ and ‘frontier understanding of industry dynamic’ should be included in the dimension of ‘Service skills’ and ‘Professionalism’, respectively.

Based on the experts’ comments, because the concept of the two items ‘Pursuit of progress’ and’ Learning and development’ in the dimension of ‘Professionalism’ was relatively repetitive, these two items should be merged into one item as ‘Pursuit of progress and development’.

The experts suggested that the item ‘achievement oriented’ was not the necessary element of the competences for family physicians; furthermore, it was considered a difficult concept to evaluate. Therefore, it was excluded from the competency model. Table 1 shows the competency model, which contains a total of 35 competency elements and definitions from four dimensions for Chinese family physicians.

**Table 1:** Key elements for the evaluation of family physician service competence under the background of Internet Plus

Dimensions	Elements	Definition
1. Service skills	1a. General medical service	Basic general clinical medical expertise and skills in diagnosis and treatment
	1b. Health management service for special population	Healthcare knowledge and skills in health management and holistic health services for children, women, the elderly, and patients with chronic diseases in the community
	1c. Health education service	Education skills with knowledge of disease prevention and health promotion
	1d. Management of infectious patients	Skills in managing people with infectious diseases such as tuberculosis and AIDS in the community
	1e. Rehabilitation service	Skills in developing the rehabilitation medical plan and providing rehabilitation knowledge for patients with physical impairments in the community
	1f. Disease surveillance	Ability to continuously monitor the high-risk population for specific diseases (e.g. hypertension/diabetes/asthma/influenza) in the community
	1g. Referral coordination	Coordinated ability to make accurate referrals to level 2 and 3 hospitals based on the patient’s condition and the expertise of the superior hospital specialist
	1h. Using telemedicine system	Skills in handling equipment such as telemedicine
	1i. Using two-way referral information system	Proficiency in the use of a two-way referral information system
	1j. Online health education	Skills in educating patients and their families through information technology tools such as apps
	1k. Online health promotion	Skills in helping patients manage their own health using information technology tools such as apps
	1l. Online health guide	Skills in providing health guidance for patients and their caregivers using the Internet
	1m. Online information exchange	Ability to use the Internet to exchange information
	1n. Understanding and implementation of primary healthcare regulations	Achieve in their efforts to ensure that they are aware of and take steps to comply with relevant laws, policies, and regulations
2. Professionalism	2a. Frontier understanding of industry dynamic	Accurate and timely understanding of the latest developments of and concepts related to Internet Plus family doctor
	2b. Professional interest and recognition	Have a strong interest and enthusiasm in the work of family physicians and be proud of this work
	2c. Industry self-discipline	Standardize and regulate your own behaviour and do not violate industry or work rules and regulations
	2d. Pursuit of progress and development	Work hard, do not be satisfied with the status quo, and actively learn new knowledge and skills to help yourself improve and develop
	2e. Spirit of service	Have the will and determination to serve patients in the community and protect the health of residents
	2f. Respect the patient’s right to know and privacy	Protect patient privacy and respects the right of patients and their families to know about their conditions
	2g. Collect and process nformation	Have the ability to collect work-related information (the health problems of the local community and other community-based resources and services) promptly and to accurately analyse and process the information
	2h. Work under stress	Effectively relieve stress and handle work when facing external pressures and setbacks
	2i. Time management	Have the ability to rationally allocate and utilize your time to effectively achieve the goals
3.Interpersonal communication and teamwork skills	3a. Communicate with patients	Fully communicate with patients to understand their personal, family, community, and social background
	3b. Doctor-patient decision-making	Promote and encourage patients’ motivation and initiative and empower patients and their families to participate in and develop treatment plans for self-health needs
	3c. Meet the needs of patients	Have the ability to discover the psychological state and needs of patients and residents
	3d. Team communication and collaboration	Communicate with, understand, and support team members to serve the team’s goals together
	3e. Actively seek help	Seek help and promptly solve problems that cannot be solved independently at work
	3f. Organizational and coordination capabilities	Have the ability to allocate resources according to work tasks, and control and coordinate normal operation of activities
4. Personal traits	4a. Patience	Patiently respond to the questions and requirements from patients and residents, and resolve them actively
	4b. Empathy	Understand the feelings of others, think and deal with the problems from the perspective of patients and residents, and be willing to take the time to care for and understand the patients
	4c. Grittiness	The family physician has perseverance and passion for long-term goals
	4d. Influence	Have the ability to use professional knowledge and facts to persuade others and influence their views or decisions
	4e. Responsibility	Have a sense of responsibility
	4f. Decisiveness	Quickly make judgments and make correct decisions about what happens at work

### Respondents and questions

A total of 450 family physicians were surveyed, with a 93.36% response rate. Eight invalid questionnaires (missing information or incomplete) were excluded. The effective rate of the questionnaire was thus 98.22%. A descriptive analysis of the characteristics of respondents was carried out: 76% were women, 55.4% were in the 30–39 age group, 79% had a bachelor’s degree, 62.2% had worked for more than 10 years, and 68% had a deputy senior title or higher. In China, the professional titles of doctors are junior (physician, physician/resident), intermediate (attending physician), deputy senior (deputy chief physician), and senior (chief physician).

Most of the respondents completed the questionnaire within 15 min, with an average completion time of 8 min. The average answer score of the 35 statements from the 442 subjects was 3.70 (SD = 0.84). Of these, the rate of strongly disagree ranged from 0.90 to 5.66%, whereas the rate of strongly agree ranged from 8.82 to 31.90% (Table 2).

**Table 2:** Family physician competency model questions and response characteristics (*n* = 442)

Items	Mean	SD	Strongly disagree (%)	Strongly agree (%)
1a. General medical service	3.96	0.84	1.81	15.16
1b. Health management service for special population	3.88	0.85	2.04	15.84
1c. Health education service	3.82	0.73	2.26	14.71
1d. Management of infectious patients	3.79	0.71	2.94	14.93
1e. Rehabilitation service	3.58	0.84	3.39	16.29
1f. Disease surveillance	3.55	0.65	3.85	13.57
1g. Referral coordination	3.71	0.84	2.49	14.48
1h. Using telemedicine system	3.75	0.76	2.71	15.16
1i. Using two-way referral information system	3.59	0.84	2.04	15.38
1j. Online health education	4.17	1.26	1.36	27.83
1k. Online health promotion	3.56	0.86	3.62	16.52
1l. Online health guide	4.18	1.36	1.13	31.90
1m. Online information exchange	3.39	0.78	4.75	14.48
1n. Understanding and implementation of regulations	3.37	0.75	5.20	13.57
2a. Frontier understanding of industry dynamic	3.41	0.71	4.30	13.35
2b. Professional interest and recognition	3.42	0.74	4.52	14.48
2c. Industry self-discipline	4.32	1.15	0.90	30.77
2d. Pursuit of progress and development	3.54	0.77	3.39	12.44
2e. Spirit of service	3.77	0.76	2.71	13.35
2f. Respect the patient’s right to know and privacy	3.80	0.75	2.71	14.03
2g. Collect and process information	3.72	0.72	2.49	12.90
2h. Work under stress	3.26	0.69	5.66	12.44
2i. Time management	3.74	0.76	4.75	15.84
3a. Communicate with patients	4.11	1.18	1.58	30.32
3b. Doctor-patient decision-making	3.61	0.80	3.17	15.16
3c. Meet the needs of patients	3.75	0.76	3.62	16.06
3d. Team communication and collaboration	3.72	0.76	2.49	14.93
3e. Actively seek help	3.21	0.77	5.43	8.82
3f. Organizational and coordination capabilities	3.65	0.81	3.17	12.67
4a. Patience	3.68	0.79	3.39	13.57
4b. Empathy	3.62	0.82	2.94	12.22
4c. Grittiness	4.11	1.16	2.04	28.96
4d. Influence	3.71	0.98	4.07	20.81
4e. Responsibility	3.75	0.84	3.17	18.10
4f. Decisiveness	3.23	0.74	4.98	12.44

### Reliability and validity analysis

The reliability analysis of the scale data was carried out with all 35 service competence evaluation items included. The Cronbach’s *α* coefficient was 0.977, indicating that the scale was very credible. The factor analysis of the scale showed that the KMO value was high, at 0.988, indicating many common factors among the variables. According to Bartlett’s spherical test, the *χ*^2^ value was 22 917.515 (df = 630), *p* < 0.001, suggesting common factors among the correlation matrices, making the scale suitable for factor analysis. The survey scale thus exhibited high structural validity.

Correlations between each of the 35 items and their theorized domain based on the initial family physician competency model were all stronger than correlations between each individual item and the other three domains.

The content validity of the scale was judged according to the Delphi method. The overall authority grade of expert consultation was 0.80, and the response rates of the two rounds of consultation were 93.3% and 86.7%, respectively. Kendall’s coefficient of concordance *W* was 0.194 (*χ*^2^ = 222.749, *p* ≤ 0.001). These results indicated that the scale demonstrated good content validity.

### Exploratory factor analysis

In this stage, 221 of the 442 respondents were randomly assigned to the EFA group. According to the results for model fit, the four-factor model showed the best fit index; however, its factor structure was chaotic, and only one item was loaded over factor four. The choice of three- or five-factor model was therefore more appropriate from the perspective of structural simplicity. In addition, from the perspective of model fit index, it was more appropriate to select a four- or five-factor model. Five-factor model was retained after comprehensive consideration.

**Fig. 1:**
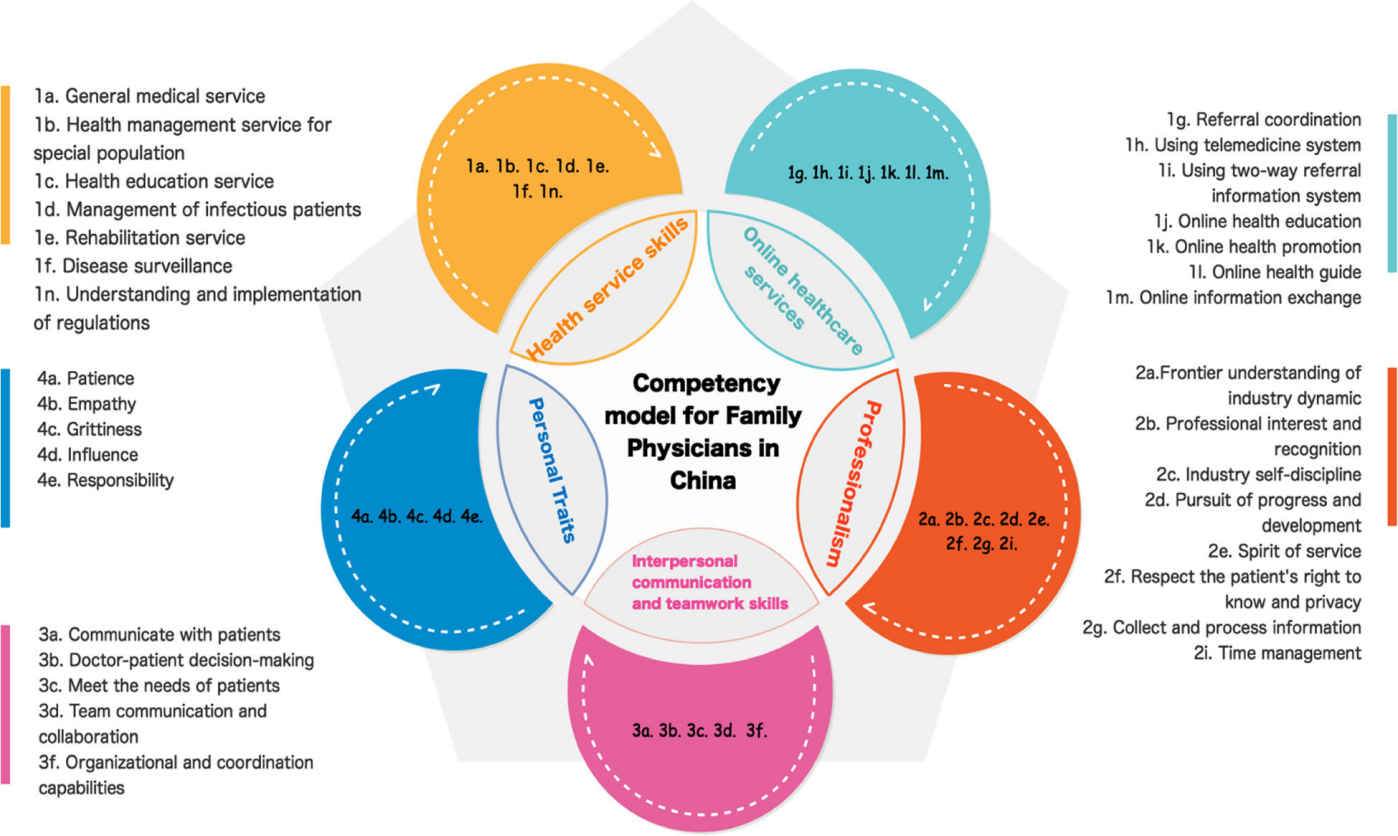
A diagram of competency model for family physicians in China. The code represents the meaning of the items specified in Table 5

The item combination of the five-factor model was different from that of the original competency model (Fig. 1). We labelled factor 1 as ‘Health service skills’ (items 1a, 1b, 1c, 1d, 1e, 1f, 1n); factor 2 as ‘Online healthcare services’ (items 1g, 1h, 1i, 1j, 1k, 1l, 1m); and factor 3 including eight items (items 2a, 2b, 2c, 2d, 2e, 2f, 2g, 2i) in the original dimension ‘Professionalism’ named ‘Professionalism’. In addition, factor 4 (including items 3a, 3b, 3c, 3d, 3f ) and factor 5 (including items 4a, 4b, 4c, 4d, 4e) were basically consistent with the dimension ‘Interpersonal communication and teamwork skills’ and ‘Personal Traits’, and hence, the two factors were named ‘Interpersonal communication and teamwork skills’ and ‘Personal Traits’, respectively.

After the exploratory factor analysis, five common factors were extracted including 32 evaluation items, which deviated from the hypothesis model (Table 3). Therefore, the proposed initial hypothesis model was revised based on the analysis results. The internal consistency test was performed on the extracted common factors, which showed that the internal consistency coefficients of the five common factors were higher than 0.80, indicating that the evaluation model had high reliability (Table 4).

**Table 3:** Exploratory factor analysis loadings of family physician competency model

Items	EFA factors (*n* = 221)				
	Factor 1	Factor 2	Factor 3	Factor 4	Factor 5
1a. General medical service	0.504*	− 0.018	0.496*	− 0.010	− 0.009
1b. Health management service for special population	0.643*	0.187	0.268	0.013	− 0.048
1c. Health education service	0.668*	0.531*	− 0.022	0.104	− 0.107
1d. Management of infectious patients	0.520*	0.487*	0.089	0.219	0.012
1e. Rehabilitation service	0.690*	0.105	0.074	0.333*	0.143
1f. Disease surveillance	0.773*	0.162	− 0.006	0.343*	0.135
1g. Referral coordination	0.462*	0.556*	0.261	− 0.009	0.141
1h. Using telemedicine system	0.291*	0.482*	0.219	− 0.048	0.158
1i. Using two-way referral information system	0.197*	0.731*	0.075	− 0.107	0.008
1j. Online health education	0.027	0.905*	− 0.085	0.012	− 0.009
1k. Online health promotion	0.127	0.836*	0.021	0.143	− 0.016
1l. Online health guide	− 0.052	0.965*	− 0.043	0.135	− 0.157*
1m. Online information exchange	− 0.025	0.987*	− 0.056	0.14	− 0.109
1n. Understanding and implementation of regulations	0.976*	0.049	− 0.033	0.158	− 0.064
2a. Frontier understanding of industry dynamic	− 0.097	0.043	0.414*	0.008	− 0.108
2b. Professional interest and recognition	− 0.055	0.068	0.868*	− 0.009	− 0.012
2c. Industry self-discipline	0.029	0.201	0.688*	− 0.016	0.013
2d. Pursuit of progress and development	− 0.081	0.330*	0.343*	0.382*	0.104
2e. Spirit of service	0.006	0.105	0.760*	0.159	0.219
2f. Respect the patient’s right to know and privacy	− 0.005	− 0.167*	0.712*	− 0.084	0.333*
2g. Collect and process information	− 0.109	− 0.004	0.921*	0.093	0.343*
2h. Work under stress	− 0.104	0.214*	0.236*	0.289*	0.372*
2i. Time management	− 0.1	0.029	0.999*	− 0.025	0.115
3a. Communicate with patients	0.12	0.058	− 0.069	0.796*	− 0.133
3b. Doctor-patient decision-making	0.085	− 0.007	0.042	0.822*	0.266*
3c. Meet the needs of patients	0.045	− 0.049	0.026	0.866*	− 0.054
3d. Team communication and collaboration	0.091	0.049	0.382*	0.804*	0.159*
3e. Actively seek help	− 0.144	− 0.005	0.159	0.159*	0.066
3f. Organizational and coordination capabilities	− 0.001	0.053	− 0.084	0.897*	0.472*
4a. Patience	0.050	− 0.072	0.093	0.115	0.848*
4b. Empathy	0.035	− 0.019	0.289*	− 0.130	0.881*
4c. Grittiness	− 0.066	0.064	− 0.025	0.066	0.902*
4d. Influence	− 0.109	0.073	0.058	0.266*	0.945*
4e. Responsibility	0.05	0.036	− 0.004	− 0.054	0.290*
4f. Decisiveness	0.104	0.108	0.079	0.159*	0.119*

*p < 0.05

**Table 4:** Fitting information indicators for EFA model

Factor	***χ***^**2**^	***df***	AIC	BIC	SRMR	RMSEA(90% CI)
Two-factor	1000.805*	492	8256.088	8969.702	0.022	0.068(0.062, 0.074)
Three-factor	1156.078*	525	8523.641	9125.116	0.027	0.074(0.068, 0.080)
Four-factor	2163.850*	594	10 128.478	10 495.480	0.069	0.109(0.104, 0.114)
Five-factor	1410.474*	559	8907.160	9393.098	0.038	0.083(0.078, 0.088)

*AIC* Akaike information criterion, *BIC* Bayesian information criterion, *SRMR* standardized root–mean–square residual, *RMSEA* root–mean–square error of approximation

*p < 0.05

### Confirmatory factor analysis

In this study, the remaining 221 samples were randomly selected for EFA models for CFA. The endogenous latent variables (service competences) was influenced by exogenous latent variables F1, F2, F3, F4, and F5 while these exogenous latent variables were measured by endogenous observational variables such as items 1a–4e (Table 5). According to the fit results, all fit indexes suggested that the model passed the goodness-of-fit test (non-normed fit index = 0.98, Tucker–Lewis index = 0.91, SRMSR = 0.05, and RMSEA = 0.04). According to the analysis results, the Cronbach’s *α* coefficients for the scale and each sub-item were greater than 0.9 (F1, Health service skills *α* = 0.91; F2, Online healthcare services *α* = 0.94; F3, Professionalism *α* = 0.92; F4, Interpersonal communication and teamwork skills *α* = 0.91; Factor 5, Personal traits *α* = 0.92), indicating that the data were reliable.

**Table 5:** Factor loading estimates for CFA model and weights

Factors	Items	Standardized factor load	S.E.	Absolute influence coefficient
Factor 1. Health service skills	1a. General medical service	0.913	0.034	0.85
	1b. Health management service for special population	0.981	0.027	0.82
	1c. Health education service	0.82	0.023	0.77
	1d. Management of infectious patients	0.852	0.02	0.79
	1e. Rehabilitation service	0.876	0.017	0.82
	1f. Disease surveillance	0.891	0.015	0.83
	1n. Understanding and implementation of regulations	0.815	0.024	0.76
Factor 2. Online healthcare services	1g. Referral coordination	0.862	0.018	0.8
	1h. Using telemedicine system	0.85	0.02	0.79
	1i. Using two-way referral information system	0.866	0.018	0.81
	1j. Online health education	0.827	0.022	0.77
	1k. Online health promotion	0.814	0.024	0.76
	1l. Online health guide	0.836	0.021	0.78
	1m. Online information exchange	0.819	0.023	0.76
Factor 3. Professionalism	2a. Frontier understanding of industry dynamic	0.837	0.021	0.78
	2b. Professional interest and recognition	0.828	0.022	0.77
	2c. Industry self-discipline	0.84	0.02	0.78
	2d. Pursuit of progress and development	0.85	0.019	0.79
	2e. Spirit of service	0.91	0.012	0.85
	2f. Respect the patient’s right to know and privacy	0.898	0.014	0.84
	2g. Collect and process information	0.874	0.017	0.81
	2i. Time management	0.912	0.012	0.85
Factor 4. Interpersonal communication and teamwork skills	3a. Communicate with patients	0.909	0.012	0.85
	3b. Doctor-patient decision-making	0.924	0.011	0.72
	3c. Meet the needs of patients	0.895	0.014	0.69
	3d. Team communication and collaboration	0.921	0.011	0.71
	3f. Organizational and coordination capabilities	0.943	0.008	0.73
Factor 5. Personal traits	4a. Patience	0.928	0.01	0.72
	4b. Empathy	0.86	0.018	0.67
	4c. Grittiness	0.926	0.01	0.72
	4d. Influence	0.86	0.018	0.67
	4e. Responsibility	0.88	0.016	0.68

## Discussion

According to the results of the study, the Family Physicians’ Service Competences Assessment Scale performed well on acceptability, validity, and reliability. Therefore, it can be considered a reliable, effective, scientific tool to comprehensively assess the service competences of Chinese family physicians in the developed urban regions at the present stage.

The final version of the scale has a total of 32 items covering five dimensions: Health service skills, Professionalism, Interpersonal communication and teamwork skills, and Personal traits. Although this deviates from the initially hypothesized model, the five-dimensional evaluation scale is more concise than the original model and still conforms to the theoretical framework established through literature review, BEIs, and expert consultation. For example, factor 3, Professionalism; factor 4, Interpersonal communication and teamwork skills; and factor 5, Personal traits, in the theoretical framework remain unchanged, and although theoretical factor 1, Service skills, were divided into two factors, ‘Health services skills’ and ‘Online healthcare services’, the original items were retained basically. Furthermore, with the wide application of information technology in the medical field, this change may emphasize the importance of online service skills for family physician competences.

To date, the international research on the definition of the service competences of family physicians has mainly consisted of the 10-dimension framework of Gay, the seven-dimension framework of the WHO, and the six-dimension framework of WONCA [24, 25]. It was well documented that the demands and challenges for family physicians while working in new models of primary care, which pursue ambitious goals to deliver timely, high-quality, well-coordinated, patient-centred care. The core elements of family physicians’ service competences highlighted in the other studies [21–25] are basically covered in our 32-item scale and include patient-centred care, health promotion, disease prevention, teamwork, and referral coordination. In addition, we have included some competency evaluation elements for family physicians who use the Internet and information technology to provide services to patients, in accordance with the actual situation in China (such as, skills in using the remote medical system; two-way referral information system; and Internet Plus approaches such as apps to educate patients and their families with health knowledge; developing Internet Plus medical treatment to help patients’ self-management; providing guidance on rehabilitation for patients and their caregivers using the Internet; and having accurate and timely understanding of the latest developments and related concepts for Internet Plus general practitioners). Some studies have shown that in recent years, information technology has become more and more widely used in medical services, especially family doctor services [33, 34]. With the rapid development of information technology, mastering the relevant information technology knowledge to provide higher-quality services for residents is a requirement and challenge that family physicians have to face. Moreover, distinct from other studies [21–23], our study has added the dimension of ‘Personal traits’ to family physicians’ service competences. Numerous studies have suggested that personal traits of doctors, such as patience and empathy, have a significant influence on the quality of medical services and patient outcomes [35–37]. Therefore, our scale more comprehensively assesses the elements of family physicians’ service competences than previous tools.

Being consistent with other previous studies [21–23], our study employed mixed methods (including literature review, BEIs, questionnaire, and expert consultation) to construct a general competency model of Chinese family physicians. We collected, integrated, and mixed both quantitative and qualitative data. The quantitative and qualitative methods complemented each other, ensuring the results reliable, credible, and rigorous.

There are some limitations to the study. We have chosen family physicians instead of patients as the respondents for our investigation of the importance of the items in the scale. The purpose of our research is to make a scientific evaluation of family physicians’ service competences and give timely feedback to the physicians so that they can tailor and achieve their own career development plans. We believe that family physicians are more comprehensively aware of all aspects of their work than patients, so we have chosen family physicians alone as the respondents. Our results show that family physicians tend to be more positive and affirmative (average scores of 3.70 out of 5 for all items), which is also known as positive skewness [38]. This shows that family physicians have a high acceptance of the various elements of the service competences assessment scale. However, previous studies have shown that when using a scale to evaluate the performance or ability of a family doctor, the doctor’s self-evaluation is less adequate than multi-dimensional evaluation from peers, co-workers, and patients [16, 39]. Therefore, the next step in the research will be to conduct an in-depth study integrating the assessment of family physicians’ service competences from peers, co-workers, and patients into the scale validation. Future research could also consider associations between Family Physicians’ Service Competences Assessment Scale measure completed by patients and family physicians. We could have the opportunity to consider what lies behind the different perceptions held by patients and family physicians and to train family physicians to improve community health service quality in a targeted way. The study findings only relate to Zhejiang province and Guangzhou province and may not be generalizable to less developed, rural areas. However, as two of the earliest health reform pilot cities, Hangzhou and Shenzhen have developed a relatively mature family doctor system. Therefore, our findings provide a snapshot of the cultivation and improvement patterns of family physicians in the context of an intensive healthcare reform. More empirical work will be required to improve this instrument by confirming scale reliability in a second sample and by conducting surveys in more regions.

It is of great importance to understand how the general medicine/family medicine discipline is evolving as the healthcare systems in which it operates evolve. To meet the growing and diverse needs for higher quality of health services of patients, family physicians must be involved in the continuous development of their healthcare system and must be able to improve their competences in order to meet these new challenges. Therefore, scales for the assessment of family physicians’ service competences should be adjusted and improved in a timely manner according to changes in family doctor service content and service model, so as to effectively improve family doctor service quality.

## Conclusions

The Family Physicians’ Service Competences Assessment Scale validated in this study, with 5 dimensions and 32 items, is the first scale instrument in China to evaluate current family physicians’ service competences against the background of the widespread use of Internet technology in primary healthcare services. The scale is proven here to be reliable, valid, and credible and provides a good tool to assess the service competences of Chinese family physicians in the developed urban regions. This study can serve as a foundation of and starting point for research on strategy to improving service competences of Chinese family physicians and also of the quality of primary care services.

## Data Availability

Data supporting the results reported in the article are available from the corresponding author upon reasonable request.

## Abbreviations

BEIs: Behaviour events interviews
EFA: Exploratory factor analysis
CFA: Confirmatory factor analysis
WONCA: World Organization of Family Doctors
SRMSR: Standardized Root Mean Square Residual
RMSEA: The root–mean–square error of approximation
NFI: Non-normed fit index
AIC: Akaike information criterion
BIC: Bayesian information criterion
